# Impact of gut hormone FGF-19 on type-2 diabetes and mitochondrial recovery in a prospective study of obese diabetic women undergoing bariatric surgery

**DOI:** 10.1186/s12916-017-0797-5

**Published:** 2017-02-16

**Authors:** Lucia Martinez de la Escalera, Ioannis Kyrou, Jana Vrbikova, Voitech Hainer, Petra Sramkova, Martin Fried, Milan K. Piya, Sudhesh Kumar, Gyanendra Tripathi, Philip G. McTernan

**Affiliations:** 1 Warwick Medical School, University of Warwick, Coventry, UK; 2 Warwickshire Institute for the Study of Diabetes, Endocrinology and Metabolism (WISDEM), University Hospitals of Coventry and Warwickshire (UHCW) NHS Trust, Coventry, UK; 3 Aston Medical Research Institute, Aston Medical School, Aston University, Birmingham, UK; 4 Institute of Endocrinology, Prague, Czech Republic; 5 OB Clinic, Prague, Czech Republic; 6 Derby Teaching Hospitals NHS Foundation Trust, Derby, UK; 7 Department of Biomedical Sciences, University of Westminster, London, W1W 6UW UK; 8 School of Science and Technology, Nottingham Trent University, Nottingham, NG11 8NS UK

**Keywords:** Gut hormone, FGF-19, Mitochondria, Type-2 diabetes recovery, Obesity, Bariatric surgery

## Abstract

**Background:**

The ileal-derived hormone, fibroblast growth factor 19 (FGF-19), may promote weight loss and facilitate type-2 diabetes mellitus remission in bariatric surgical patients. We investigated the effect of different bariatric procedures on circulating FGF-19 levels and the resulting impact on mitochondrial health in white adipose tissue (AT).

**Methods:**

Obese and type-2 diabetic women (*n* = 39, BMI > 35 kg/m^2^) undergoing either biliopancreatic diversion (BPD), laparoscopic greater curvature plication (LGCP), or laparoscopic adjustable gastric banding (LAGB) participated in this ethics approved study. Anthropometry, biochemical, clinical data, serum, and AT biopsies were collected before and 6 months after surgery. Mitochondrial gene expression in adipose biopsies and serum FGF-19 levels were then assessed.

**Results:**

All surgeries led to metabolic improvements with BPD producing the greatest benefits on weight loss (↓30%), HbA1c (↓28%), and cholesterol (↓25%) reduction, whilst LGCP resulted in similar HbA1c improvements (adjusted for BMI). Circulating FGF-19 increased in both BPD and LGCP (χ^2^(2) = 8.088; *P* = 0.018), whilst, in LAGB, FGF-19 serum levels decreased (*P* = 0.028). Interestingly, circulating FGF-19 was inversely correlated with mitochondrial number in AT across all surgeries (*n* = 39). In contrast to LGCP and LAGB, mitochondrial number in BPD patients corresponded directly with changes in 12 of 14 mitochondrial genes assayed (*P* < 0.01).

**Conclusions:**

Elevated serum FGF-19 levels post-surgery were associated with improved mitochondrial health in AT and overall diabetic remission. Changes in circulating FGF-19 levels were surgery-specific, with BPD producing the best metabolic outcomes among the study procedures (BPD > LGCP > LAGB), and highlighting mitochondria in AT as a potential target of FGF-19 during diabetes remission.

## Background

A key factor in the development of type-2 diabetes and metabolic syndrome is the inability of adipose tissue (AT) to cope with the chronic insult of over-nutrition, whilst maintaining important metabolic and endocrine functions [1, 2]. At the forefront of this challenging environment are mitochondria, major nutrient sensors and metabolic regulators, which are fundamental to AT function [3, 4]. However, during sustained conditions of chronic nutrient excess, such as obesity and type-2 diabetes, mitochondria appear unable to cope well with this environment, leading to fragmentation, unresponsiveness and dysfunction [5–7]. This nutrient-induced mitochondrial dysfunction can lead to impaired respiration, lipotoxicity, oxidative species accumulation and inflammation, further exacerbating insulin resistance and type-2 diabetes [8–13]. Indeed, the importance of adequate mitochondrial function for metabolic health is further highlighted by the observation that mitochondrial DNA mutations often result in diabetic phenotypes [14–16].

Both insulin resistance and type-2 diabetes status can be reversed through bariatric surgery, with significantly greater success rates than pharmacological, exercise and diet interventions [17–19]. Depending on the procedure, bariatric surgery involves a type/degree of gastro-intestinal remodelling, which can lead to reduced stomach volume and nutrient absorption capacity [20, 21]; however, this alone cannot fully explain the profound weight loss and metabolic improvement observed after these surgeries versus medical/lifestyle interventions [22].

Recently, the ileal-derived hormone, fibroblast growth factor 19 (FGF-19), has been identified as a novel enterokine regulator of glucose and lipid homeostasis, which is potentially involved in metabolic recovery following bariatric surgery [23]. Indeed, rodent studies have shown that mice lacking the receptor required for gut secretion of FGF-19 show significantly impaired weight loss and glucose improvement following bariatric surgery compared with their wild-type counterparts [24]. In addition, direct administration of recombinant FGF-15 (FGF-19 in humans) to obese mice leads to significant weight reduction, principally AT reduction, and reverses dietary and leptin-deficient diabetes [25]. Moreover, in humans, data from clinical studies would seemingly indicate FGF-19 as a cause rather than consequence of type-2 diabetes improvement, given that neither lifestyle interventions nor intense medical management of type-2 diabetes appears to increase circulating FGF-19 levels, despite similar reductions in HbA1c to surgical procedures [26]. However, there is good clinical evidence that certain bariatric procedures increase serum FGF-19 levels [27–30]. As such, both human and rodent studies suggest that increased circulating FGF-19 levels may contribute to the underlying mechanisms of metabolic improvement following certain types of bariatric surgery.

Beyond the potential effects on white AT, studies have shown FGF-19 to exert several advantageous effects on various metabolic relevant organs [23]. In the central nervous system, FGF-19 has been associated with lowered brain-hedonistic responses, reduced food intake, improved glycaemic control and enhanced glucose effectiveness [31, 32]. Furthermore, in the liver, FGF-19 has been shown to increase energy expenditure and fatty acid oxidation through raised delivery of fatty acids to the mitochondria [33]. Additionally, in brown AT, elevated FGF-19 (either through genetic over-expression or systemic administration) can affect the metabolic rate and activity of this highly energy-consuming tissue [25, 33]. These studies also stress the importance of mitochondria as a target of FGF-19 [25, 31–33], although its role in white AT mitochondria, particularly within the context of type-2 diabetes, remains largely unknown. Therefore, in the present study, we investigated the hypothesis that changes in serum FGF-19 levels after bariatric surgery support metabolic recovery via improvement in mitochondrial function within white AT.

## Methods

### Ethics and study design

The study was approved by the Ethics Committee of the Institute of Endocrinology (Institute of Endocrinology, Ethics Committee EC: 19/5/2009, Prague, Czech Republic). All study participants provided written and informed consent in accordance with the Declaration of Helsinki. Thirty-nine morbidly obese (BMI > 35 kg/m^2^), type-2 diabetic, Caucasian women undergoing either biliopancreatic diversion (BPD; *n* = 12), laparoscopic greater curvature plication (LGCP; *n* = 15), or laparoscopic adjustable gastric banding (LAGB; *n* = 12) at the OB clinic, Prague, Czech Republic, were recruited to participate in this study. Thorough biochemical and anthropometric investigations were conducted before (baseline) and at 6 months after surgery with collection of serum samples and abdominal subcutaneous white AT biopsies at both of these time points. Patients on pharmacological treatment with incretin mimetics and/or insulin were not included in this study.

### Blood biochemistry and body composition analysis

All anthropometric and biochemical measurements were performed before and 6 months after surgery. Following a 10-hour overnight fast, venous blood was sampled in all patients, collected in chilled EDTA-containing tubes with and without aprotinin (for glucose and insulin measurements), aliquoted and frozen at –80 °C until assayed. Serum glucose, HbA1c and lipids were determined using the Cobas 6000 analyzer. Insulin resistance was assessed using the homeostatic model assessment of insulin resistance (HOMA-IR) according to the following equation: HOMA-IR = fasting glucose (mmol/L) × fasting insulin (mIU/L)/22.5, as previously described [34]. The Friedwald formula [35] was employed to compute serum levels of LDL cholesterol. Body weight was measured to the nearest 0.5 kg and height to the nearest 1 cm. Percentage excess weight loss was calculated according to the following equation: (preoperative weight-postoperative weight/preoperative weight-ideal body weight) × 100, and body fat mass was measured using the bioimpedance method (Tanita TBF-300; Tanita corporation).

### RNA isolation and qRT-PCR

For RNA extraction, 100 mg of frozen AT was homogenized in 500 μL Qiazol reagent (#79306 Qiagen, UK) then isolated using a column-based isolation method (RNeasy Lipid Tissue Mini Kit; #74804 Qiagen, UK) according to manufacturer’s instructions. Samples were digested with DNase I to remove potential genomic DNA contaminants (DNase I kit, #AMP-D1 Sigma-Aldrich). RNA was eluted in 10 μL RNase-free water and 1 μL quantified in duplicate using a spectrophotometer (Nanodrop ND-1000, labtech) at 260 nm absorbancy. Synthesis of cDNA was performed using 200 ng RNA per sample and a Bioline mRNA reverse transcription kit (#BIO-65026) according to the manufacturer’s instructions. Gene expression was assayed through quantitative real-time polymerase chain reaction (qRT-PCR) using ABI 7500 standard sequence detection system (Applied Biosystems, UK). Each reaction was prepared to 25 μL final volume containing Taqman Universal PCR mastermix (#4304437 Applied Biosystems, UK), 1 μL sample cDNA and a specific commercially available Taqman gene expression assay (Applied Biosystems, UK; *PGC1α*, Hs00173304_m1; *POLG*, Hs01018668_m1; *TFAM*, Hs00273372_s1; *mtND6*, Hs02596879_g1; *SDHA*, Hs00188166_m1; *COX4I1*, Hs00971639_m1; *mtATP6*, Hs02596862_g1; *UCP2*, Hs01075227_m1; *SOD1*, Hs00533490_m1; SOD2, Hs00167809_m1; MFN2, Hs00208382_m1; OPA1, Hs01047018_m1; DRP1, Hs01552605_m1; FIS1, Hs00211420_m1). All samples were assayed in triplicate and multiplexed using 18S (ribosomal RNA) as a pre-optimised control probe. As per the manufacturer’s instructions, reactions were carried out at 50 °C for 2 minutes, 95 °C for 10 minutes, and then 40 cycles of 95 °C for 15 seconds and 60 °C for 1 min. For data analysis, a ΔCt was calculated based on the difference between 18S and the target gene. Gene expression was calculated based on the following formula: mRNA expression = 2^–ΔΔCt^, where ΔCt = *target gene – 18S.*

### Evaluation of mitochondrial number

Total DNA was extracted from 50 mg frozen AT samples using DNeasy Blood and Tissue Mini Kit (#69504 Qiagen, UK) in accordance to the manufacturer’s instructions. RNase treatment was performed to eliminate possible RNA contamination. DNA was eluted with 100 μL AE buffer and quantified using a spectrophotometer (Nanodrop ND-1000, Labtech). Relative amounts of mitochondrial DNA copy number were assessed through qPCR in an ABI Prism 7500 thermo cycler (Life Technologies) with the use of iQ™ SYBR Green Supermix (#170-8880 BioRad). Mitochondrial (*mtND1*; forward: *5’-ATGGCCAACCTCCTACTCCT-3’*; reverse: *5’-GCGGTGATGTAGAGGGTGAT-3’*) and nuclear (*BECN1*; forward: 5’-*CGAGGCTCAAGTGTTTAGGC-3’*; reverse: *5’-ATGTACTGGAAACGCCTTGG-3’*) gene primers were used to determine relative amounts of mitochondrial to nuclear DNA [36]. Each sample was measured in triplicate. Mitochondrial number was calculated based on the following formula: mtDNA copy number = 2^ΔCt^, where ΔCt = *BECN1* – *mtND1*.

### FGF-19 serum levels

For measurement of serum FGF-19 levels (pg/mL), an enzyme-linked immunosorbent assay (ELISA) kit for FGF-19 (Quantikine ELISA, R&D Systems, Minneapolis, MN) was used. All measurements were performed in duplicate according to the manufacturer’s instructions. This assay has a detection range of 31–544 pg/mL and a coefficient of variation of 4.5% for intra-assay and 5.5% inter-assay precision.

### Statistical analysis

Statistical analyses were performed using the SPSS 21.0 software. Data are reported as mean ± standard deviation (SD), unless otherwise specified. Data were examined for normality according to the Shapiro–Wilks criteria. Comparisons between pre- and post-surgery time-points were performed via paired two-tailed t-tests (if parametric) and the Wilcoxon signed ranks test (if non-parametric). For categorical data, Fisher’s exact test was used. Between-group (surgery type) differences were assessed using one-way ANOVA (if parametric) and Kruskal–Wallis test (if non-parametric) using change variables, calculated as percentage change from pre-surgery values [(post/pre) × 100]. For Pearson correlation analyses, change variables [(post/pre) × 100] were log-transformed prior to analysis if non-parametric.

## Results

### BPD patients exhibited greater weight loss and improvements in serum HbA1c, total and LDL cholesterol

Clinical, anthropometric and biochemical data obtained before and 6 months after BPD (*n* = 12), LGCP (*n* = 15) or LAGB (*n* = 12) weight loss operations are shown in Table 1. All surgeries significantly improved body weight, HOMA-IR and serum HbA1c; however, BPD resulted in significantly greater reductions of excess weight loss (approx. 31%, *P* = 0.004), serum total cholesterol (24%, *P* = 0.00001) and LDL cholesterol (29%, *P* = 0.001). Serum HDL cholesterol was also significantly lower after BPD; however, the improvement in HDL/LDL ratio appeared greater with BPD (15% increase from pre-surgery, *P* = 0.154) than with LGCP and LAGB procedures (2% and 4%, respectively).

**Table 1:** Anthropometric and metabolic variables pre-surgery and 6 months after biliopancreatic diversion (BPD), laparoscopic greater curvature plication (LGCP) and laparoscopic adjustable gastric banding (LAGB) bariatric procedures

	BPD			LGCP			LAGB		
	Pre-surgery	Post-surgery	Change (%)	Pre-surgery	Post-surgery	Change (%)	Pre-surgery	Post-surgery	Change (%)
*n*	12			15			12		
Age, years	50.57 ± 5.88			53.24 ± 7.48			53.57 ± 11.26		
EWL, %	30.67 ± 8.42****			17.36 ± 6.32			20.17 ± 9.36		
Body weight, kg	127.7 ± 22.6	106.9 ± 18.5**	83.8 ± 3.0***	109.2 ± 16.5	97.6 ± 14.1**	89.5 ± 3.2	119.6 ± 19.1	105.3 ± 18.3**	88.1 ± 5.7
BMI, kg/m^2^	46.45 ± 7.75	39.01 ± 6.37**	84.1 ± 3.5***	40.41 ± 5.54	36.05 ± 4.62**	89.3 ± 3.4	43.95 ± 6.60	38.31 ± 7.05**	88.90 ± 4.9
WHR, cm	0.93 ± 0.07	0.90 ± 0.07	97.7 ± 9.9	0.88 ± 0.10	0.87 ± 0.08	100.1 ± 11.1	0.90 ± 0.04	0.88 ± 0.04	98.5 ± 5.9
Body fat, %	49.59 ± 3.70	44.68 ± 4.62**	90.2 ± 6.6	48.49 ± 3.78	44.90 ± 3.71**	92.7 ± 4.6	49.54 ± 3.44	46.39 ± 4.45*	93.6 ± 5.6
Glucose, mmol/L	8.40 ± 2.57	7.01 ± 1.73	89.1 ± 30.1	8.93 ± 2.11	7.34 ± 1.64*	84.4 ± 16.9	9.30 ± 2.65	7.07 ± 1.56**	77.4 ± 8.8
Insulin, mmol/L	31.48 ± 21.2	17.12 ± 12.0*	71.2 ± 73.8	26.43 ± 19.4	16.76 ± 10.9*	70.6 ± 24.7	26.20 ± 6.93	15.21 ± 5.92**	61.3 ± 26.1
HOMA-IR	11.8 ± 9.74	5.3 ± 4.73*	66.1 ± 97.2	10.9 ± 9.14	5.32 ± 3.34**	61.0 ± 29.5	10.8 ± 4.13	5.07 ± 2.89**	47.1 ± 20.5
HbA1c, %	7.1 ± 0.9	5.7 ± 0.70**	72.1 ± 15.1****	7.3 ± 1.0	6.5 ± 0.9**	85.1 ± 8.5	7.0 ± 0.9	6.4 ± 0.6*	89.5 ± 12.5
HbA1c, mmol/mol	53.84 ± 9.6	38.38 ± 7.8**	72.1 ± 15.1****	56.33 ± 10.5	47.93 ± 10.3**	85.1 ± 8.5	52.92 ± 10.1	46.64 ± 6.2*	89.5 ± 12.5
Total cholesterol, mmol/L	4.91 ± 1.03	3.72 ± 0.86**	75.9 ± 9.80***	4.84 ± 0.77	4.73 ± 0.82	97.8 ± 8.7	4.83 ± 0.77	4.54 ± 0.89	94.7 ± 14.9
Triglycerides, mmol/L	1.43 ± 0.66	1.59 ± 0.69	117.6 ± 51.2	1.95 ± 1.39	1.38 ± 0.71	89.2 ± 33.0	1.79 ± 0.75	1.23 ± 0.50	74.0 ± 24.4
HDL cholesterol, mmol/L	1.05 ± 0.21	0.81 ± 0.22**	73.1 ± 22.0****	1.12 ± 0.33	1.15 ± 0.31	104.0 ± 15.1	1.04 ± 0.24	1.08 ± 0.25	105.0 ± 17.3
LDL cholesterol, mmol/L	3.19 ± 1.03	2.23 ± 0.67**	71.4 ± 13.2****	2.82 ± 0.69	2.94 ± 0.77	107.2 ± 25.6	2.96 ± 0.65	2.90 ± 0.82	101.6 ± 33.4
HDL/LDL ratio	0.36 ± 0.14	0.40 ± 0.12	115.3 ± 28.6	0.42 ± 0.17	0.42 ± 0.18	102.0 ± 29.0	0.37 ± 0.11	0.38 ± 0.14	103.6 ± 25.6

Data are means ± standard deviation. Within group statistical significance (pre- to post-surgery) was determined using two-tailed paired t-test or Wilcoxon signed ranks test (*P < 0.05; **P < 0.01), whilst for between group comparisons one-way ANOVA or the Kruskal–Wallis test were used (***P < 0.05; ****P < 0.01)

^a^Denotes post-surgery values as percentage of pre-surgery values

All serum determinations correspond to fasting status

*EWL* excess weight loss, *BMI* body mass index, *WHR* waist-hip ratio, *HOMA-IR* Homeostatic assessment model of insulin resistance, *HbA1c* glycated haemoglobin

BPD patients also achieved significantly greater improvements in serum HbA1c, compared with LGCP (*P* = 0.022) and LAGB (*P* = 0.002). However, after controlling for BMI, BPD and LGCP were noted to have similar effects on HbA1c reduction, whilst the difference between BPD and LAGB remained statistically significant (*P* = 0.028).

### Post-surgery serum FGF-19 levels increased in BPD and LGCP patients, but decreased after LAGB

The majority of the BPD (58%) and LGCP (73%), but only 17% of LAGB patients exhibited increased post-surgery serum FGF-19 levels relative to pre-surgery values (Table 2). Overall, post-surgery serum FGF-19 levels in LAGB patients were significantly lower than pre-surgery values (*P* = 0.028), whilst the surgery-induced changes in FGF-19 concentrations were significantly different between the three bariatric procedures in the study (as tested using the Kruskal–Wallis H test, *P* = 0.018).

**Table 2:** Comparisons of surgery-induced changes in serum FGF-19 levels between biliopancreatic diversion (BPD), laparoscopic greater curvature plication (LGCP) and laparoscopic adjustable gastric banding (LAGB) bariatric procedures

Bariatric surgery (*n*)	Percent of patients with increase (%)	Change from pre- to post-surgery (%)^a^	
		Mean (SD)	Median (IQR)
BPD (12)	58.3	158.90 (180.60)	121.72 (52.73–152.67)
LGCP (15)	73.3	181.32 (209.65)	135.41 (74.75–172.57)
LAGB (12)	16.7	84.27 (88.49)*	63.28 (41.66–79.24)*

Table shows percentage of patients (%) who exhibited increased serum FGF-19 post-surgery relative to pre-surgery levels. The Wilcoxon signed-rank test was used for within group comparisons of pre- and post-surgery levels (*P < 0.05)

^a^The Kruskal–Wallis H test determined there were significant differences in serum FGF-19 between the three surgery types (χ^2^ = 7.655; *P* = 0.022)

*SD* standard deviation, *IQR* interquartile range

### Surgery-induced changes in serum FGF-19 levels were significantly associated with mitochondrial number in white AT

Abdominal subcutaneous white AT biopsies taken before and 6 months after bariatric surgery were used to assess the mRNA expression levels of genes involved in a wide array of mitochondrial functions (biogenesis, oxidative phosphorylation, uncoupling and antioxidant action), as well as mitochondrial number. Changes in FGF-19 levels were significantly associated with changes in adipose mitochondrial number across all surgeries (Table 3). Indeed, circulating FGF-19 was inversely correlated with mitochondrial number in AT across all surgeries (*n* = 39), suggestive of a less fragmented mitochondrial network when FGF-19 levels are elevated post-surgery. Neither FGF-19 nor AT mitochondrial number were noted to correlate significantly with any other biochemical or anthropometric parameter assessed in this study, including weight loss, BMI, HOMA-IR, serum HbA1c, or lipids.

**Table 3:** Correlations between surgery-induced changes in serum biochemical variables and mitochondrial parameters in white adipose tissue

	Correlation statistics	
	Pearson’s r	*P* value
FGF-19		
Mitochondrial number	–0.400	0.023
Total cholesterol		
mtATP6	–0.318	0.038
UCP2	–0.343	0.024
HDL cholesterol		
COX4I1	–0.335	0.030
mtATP6	–0.359	0.020

Pearson’s correlation coefficient analyses were performed using change variables (pre- to post-surgery percentage change) in the total patient cohort (*n* = 39) between serum biochemistry (bold) and mitochondrial genes

*mtATP6* mitochondria-DNA-encoded ATP synthase subunit 6 (complex V), *UCP2* uncoupling protein 2, *COX4I1* Cytochrome c oxidase subunit 4 isoform 1 (complex IV)

Of all variables captured in this study, mRNA expression of mitochondrial genes in white AT biopsies was significantly correlated only with total cholesterol and HDL cholesterol (Table 3). Indeed, post-surgery reduction in total cholesterol and HDL cholesterol levels were associated with increased expression of mitochondrial-encoded ATP synthase subunit 6 (mtATP6) and uncoupling protein 2 (UCP2), and of mtATP6 and cytochrome c oxidase subunit 4 isoform 1 (COX4I1) genes, respectively.

### Control of mitochondrial gene regulation varied with bariatric surgical procedure, with greater control observed after BPD

In order to further examine the overall impact on mitochondrial functionality in AT biopsies, surgery-induced changes in genes involved in mitochondrial function (biogenesis, oxidative phosphorylation, uncoupling, and antioxidant capacity) and dynamics (fission and fusion) were compared to the changes observed in mitochondrial number using Pearson correlation analyses. In genes controlling function, these relationships were significantly positive after BPD surgery across 9 of 10 genes assessed, whilst significantly negative for seven genes after LGCP surgery, and absent for all genes after the LAGB procedure (Table 4). Analysis of mitochondrial dynamics genes revealed significant correlations in genes involved in both fusion and fission processes within the BPD cohort. These relationships were absent in the LGCP group and present only for fusion genes in the LAGB group, indicating that the control of mitochondrial function and dynamics differed with the type of surgical procedure.

**Table 4:** Relationship of mitochondrial number to mitochondrial function and dynamics genes after biliopancreatic diversion (BPD), laparoscopic greater curvature plication (LGCP) and laparoscopic adjustable gastric banding (LAGB) bariatric procedures

	Mitochondrial number vs.	BPD (*n* = 12)	LGCP (*n* = 15)	LAGB (*n* = 12)
Function	PGC1α	0.794**	–0.688**	–0.175
	POLG	0.867**	–0.407	0.035
	TFAM	0.479	–0.560*	–0.154
	mtND6	0.758*	–0.613*	–0.153
	SDHA	0.855**	–0.600*	–0.056
	COX4I1	0.939**	–0.442	0.147
	mtATP6	0.782**	–0.547*	0.056
	UCP2	0.818**	–0.389	0.063
	SOD1	0.842**	–0.604*	0.098
	SOD2	0.696*	–0.576*	–0.017
Dynamics	MFN2	0.983**	–0.493	0.939*
	OPA1	0.808*	–0.202	0.963*
	DRP1	0.302	–0.426	0.669
	FIS1	0.871*	–0.337	0.209

Table shows Pearson’s correlation coefficient between mitochondrial number and genes involved in mitochondrial biogenesis (PGC1α, POLG, TFAM), oxidative phosphorylation (mtND6, SDHA, COX4I1, mtATP6), uncoupling (UCP2), antioxidant (SOD1, SOD2), fusion (MFN2, OPA1) and fission (DRP1, FIS1) processes. Correlations were calculated using change variables (pre- to 6-months post-surgery percentage change). *P < 0.05, **P < 0.01

*PGC1α* Peroxisome proliferator-activated receptor gamma coactivator 1-alpha, *POLG* mitochondrial DNA polymerase gamma catalytic subunit, *TFAM* mitochondrial transcription factor A, *mtND6* mitochondrially encoded NADH dehydrogenase 6, *SDHA* Succinate dehydrogenase complex subunit A, *COX4I1* Cytochrome c oxidase subunit 4 isoform 1 (complex IV), *mtATP6* mitochondria-DNA-encoded ATP synthase subunit 6 (complex V), *UCP2* uncoupling protein 2, *SOD1* superoxide dismutase 1

## Discussion

In the present study, we hypothesized that bariatric surgery-induced elevation of serum FGF-19 target mitochondrial function in white AT and support metabolic recovery. Our findings highlight for the first time (1) a direct association between FGF-19 levels and mitochondrial number in AT consistent across three surgical procedures and (2) a differential impact of certain bariatric procedures on circulating FGF-19 levels, with (3) BPD surgery leading to a tighter control of mitochondrial gene expression than LGCP or LAGB in association with greater HbA1c, lipid and weight reduction. Thus, within the post-surgery follow-up period of our study, a step-wise order in surgical benefit based on FGF-19 levels and better metabolic health outcomes was established (BPD > LGCP > LAGB).

The finding that FGF-19 levels are inversely correlated with mitochondrial number in AT may be interpreted as a shift towards a less fragmented and more elongated mitochondrial network when FGF-19 levels are raised. This would seem of benefit, given that mitochondrial fragmentation has been associated with apoptosis [37, 38], severely compromised mitochondrial DNA integrity, inefficiency [39, 40], accumulation of reactive oxygen species [6], impaired oxygen consumption and ß-oxidation [7, 8], lipotoxic species accumulation [41], pro-inflammatory cytokine production [9], and impaired insulin signalling [10, 11]. Moreover, fragmentation of muscle mitochondria has been reported in several mouse and human models of obesity and type-2 diabetes [42, 43].

However, it must also be stated that long-term sustained mitochondrial elongation can compromise mitochondrial quality control and function [44], so mitochondrial elongation per se is not necessarily indicative of mitochondrial health, and that the cell requires a balance between both fission and fusion processes to maintain mitochondrial quality. Thus, to better understand the implications of the changes observed in mitochondrial number after surgery, we analyzed them in relation to changes in mitochondrial gene expression. Genes controlling both fusion and fission processes were tightly correlated with mitochondrial number in BPD patients, whilst in the other surgeries, the genes controlling these processes (particularly fission) appeared dysregulated. In addition, following the BPD procedure alone, mitochondrial number was significantly and positively correlated with mRNA expression of most genes assayed, covering a range of mitochondrial (biogenesis, oxidative phosphorylation, uncoupling and antioxidant) functions. This finding would support the assertion that BPD improves the control of genes involved in maintaining mitochondrial fusion/fission balance and function to a greater extent than the other two bariatric procedures in this study, and is consistent with a role of serum FGF-19 in mediating a less fragmented and potentially more functional mitochondrial network.

In contrast, in the LGCP group, the relationships between mitochondrial number and gene expression followed a significant inverse association, despite similar rise in serum FGF-19 levels compared to BPD. This seemingly paradoxical finding may be better understood within a wider context of additional factors also likely to play a role in mitochondrial recovery [3]. Indeed, the BPD operation (unlike LGCP) produced significantly lower serum lipid levels and nearly twice as much weight loss (30% versus 17%). This is consistent with previous reports [45], and the notion that these two factors (weight loss and lipid recovery) may have also contributed to the enhanced mitochondrial outcomes observed after BPD versus LGCP. Further in support of this concept, total and HDL cholesterol were the only biochemical variables (apart from FGF-19) to exhibit a significant association with mitochondrial genes. Decreased cholesterol levels were directly associated with enhanced mRNA expression of complex IV (*COX4I1*) and V (*mtATP6*) genes of the electron transport chain. Similar associations were observed with the uncoupling protein 2 (*UCP2*) gene, which has been implicated in preventing reactive oxygen species accumulation and oxidative stress damage [46].

Interestingly, in the LAGB group (the only study procedure to significantly reduce serum FGF-19 levels), changes in mitochondrial gene expression in AT were (with exception of fusion genes) unrelated to mitochondrial number, suggesting a dysregulation of mitochondrial function in this cohort, potentially resulting from un-opposed fusion. Though this bariatric procedure resulted in significant weight loss and general metabolic improvement, the noted HbA1c reduction was significantly less pronounced compared with the other two procedures (even after accounting for BMI), which might be, at least in part, the result of the mitochondrial dysfunction and lower serum FGF-19 levels observed.

Previous studies in mice support the hypothesis that circulating FGF-19 targets WAT mitochondria to exert metabolic improvements. Mice challenged with a high-fat diet and treated with fexaramine (an intestine-restricted FXR agonist which potently induces intestinal FGF-15, i.e. the mouse FGF-19 homologue) exhibited significantly less weight gain, systemic inflammation and improved glucose homeostasis, with specific effects noted on visceral white AT, including reduced activation of inflammatory and lipogenic pathways, browning of white adipocytes, and increased thermogenesis [47]. Though FGF-19 is known to exert several metabolically beneficial effects by its actions in the liver that regulate glucose and cholesterol production [23], recent evidence in mice further suggests that the improvement of glucose homeostasis after recombinant FGF-15 treatment is likely due to direct signaling in AT and other metabolic relevant organs rather than through the known hepatic effects [48]. Furthermore, previous reports of positive correlations between circulating FGF-19 and adiponectin [49, 50] lend further credence to the role of FGF-19 as a regulator of WAT endocrine and metabolic function. In accordance with previous research, our findings support the hypothesis that FGF-19 targets white AT and provide evidence for the first time in humans that circulating FGF-19 levels strongly and inversely associate with mitochondrial fragmentation of this tissue.

We should note that our study has certain limitations. Firstly, though our study subjects did not follow a particular dietary regimen and led a relatively sedentary lifestyle in the period before surgery, these two factors were not controlled either before or after surgery. Secondly, despite the prospective study design, it is not possible to clarify in the context of this study the precise mechanism by which each studied surgical procedure alters serum FGF-19 levels, thus further research is required to clarify this point. However, to our knowledge, this is the first study to compare serum FGF-19 levels between these bariatric surgical procedures and to provide evidence of differential mitochondrial and metabolic outcomes based on the type of surgical procedure.

## Conclusion

In conclusion, elevated serum FGF-19 levels post-surgery were significantly associated with improved mitochondrial health in AT, leading to greater control of mitochondrial gene regulation and overall type-2 diabetes remission. These increased FGF-19 levels were also observed to be surgery-specific, with BPD patients achieving better metabolic health outcomes compared to LGCP and LAGB (BPD > LGCP > LAGB), and highlighting mitochondria in AT as a promising potential target of FGF-19 during diabetic recovery following bariatric surgery.

## Abbreviations

AT: adipose tissue
BPD: biliopancreatic diversion
*COX4I1*: cytochrome c oxidase subunit 4 isoform 1 (Complex IV)
*DRP1*: dynamin-1-like protein
*FGF-19*: fibroblast growth factor 19
*FIS1*: mitochondrial fission 1 protein
LAGB: laparoscopic adjustable gastric banding
LGCP: laparoscopic greater curvature plication
MFN2: mitofusin 2
*mtATP6*: mtDNA-encoded ATP synthase subunit 6 (Complex V)
mtDNA: mitochondrial DNA
*mtND6*: mtDNA-encoded NADH-ubiquinone oxidoreductase chain 6 (Complex II)
*OPA1*: optic atrophy 1
*PGC1α*: peroxisome proliferator-activated receptor γ coactivator 1 α
*POLG*: mitochondrial DNA polymerase gamma
*SDHA*: succinate dehydrogenase complex II subunit A
*SOD1*: superoxide dismutase 1
*TFAM*: mitochondrial transcription factor A
*UCP2*: uncoupling protein 2
